# Expert Witnesses Appointed by the Court

**Published:** 1904-07

**Authors:** 


### EXPERT WITNESSES APPOINTED BY THE COURT.

The method of appointing expert witnesses has been so much discussed pro and con in every way that court decisions which throw any light on it are welcome. In a recent case in Kentucky the appellant pleaded as an error that the two medical witnesses who testified in the case regarding the extent of the plaintiff's injuries were appointed by the court, and that the attorney for the other side had been allowed to make this fact known to the jury. The Court of Appeals decided that the physicians had been appointed by the court under the large discretion which it possessed. The question was not a matter as to how they came to know the facts, but as to the truth of their testimony. It would appear from this that a precedent has been fairly set in that State for the appointment of expert witnesses in damage cases by the court. Medical expert testimony is no worse than any other expert testimony, but it suffers by the fact that the courts usually recognize any *soidisant* expert as perfectly competent to act in that capacity, and the juries are not supposed to be discriminating as to the quality of those who thus represent themselves.—*The Journal.*

